# Submucosal Injection Using Epinephrine-Added Saline in Cold Snare Polypectomy for Colorectal Polyps Shortens Time Required for Resection: A Randomized Controlled Study

**DOI:** 10.7759/cureus.39164

**Published:** 2023-05-17

**Authors:** Atsushi Katagiri, Norihiro Suzuki, Shinya Nakatani, Kazuo Kikuchi, Takahisa Fujiwara, Toshihiko Gocho, Kenichi Konda, Kazuya Inoki, Fuyuhiko Yamamura, Hitoshi Yoshida

**Affiliations:** 1 Department of Medicine, Division of Gastroenterology, Showa University School of Medicine, Tokyo, JPN

**Keywords:** randomized controlled trial, submucosal injection, immediate bleeding, colorectal polyp, cold snare polypectomy

## Abstract

Aims: Immediate bleeding after cold snare polypectomy (CSP) for colorectal polyps might interfere with confirmation of residuals and prolong the time required for resection. We investigated whether submucosal epinephrine-added saline injection reduces the time required for the CSP procedure.

Methods: We conducted a single-center, prospective, randomized controlled trial (clinical trial registration number: UMIN000046770). Patients with colorectal polyps ≤ 10 mm were randomly allocated to either CSP with epinephrine-added submucosal injection (CEMR group) or conventional CSP (CSP group). The primary outcome was the time required for resection defined as the time from the initiation of resection (the first insertion of the snare in the CSP group or the injection needle in the CEMR group) to the end of resection (confirming complete resection endoscopically after recognizing the cessation of immediate bleeding) in each lesion, and the secondary outcome was the time to spontaneous cessation of immediate bleeding after resection defined as the time from ensnaring the lesion to confirming the spontaneous cessation of immediate bleeding.

Results: A total of 126 patients were randomly assigned. Finally, 261 lesions in 118 patients (CEMR group, n = 59; CSP group, n = 59) were analyzed. The time required for resection calculated using the least-square mean was significantly shorter in the CEMR group (106.3 s, 95% CI 97.5 to 115.4 s) than in the CSP group (130.9 s, 95% CI 121.2 to 140.7 s) (P < 0.001). The time to spontaneous cessation of immediate bleeding was also significantly shorter in the CEMR group (20.4 s, 95% CI 14.3 to 26.5 s) than in the CSP group (74.2 s, 95% CI 67.6 to 80.7 s) (P < 0.001). Neither group had cases requiring hemostasis, perforation, or delayed bleeding.

Conclusions: CEMR shortened the time for resection by shortening the time to cessation of immediate bleeding compared with conventional CSP in colorectal polyps ≤ 10 mm.

## Introduction

Colonoscopy allows effective detection and removal of precursor adenomatous polyps and is the recommended modality for optimal screening and treatment of polyps [1]. Previous studies have demonstrated that the excision of adenomatous polyps can prevent colorectal cancer (CRC) and the associated mortality [2]. Serrated polyps such as hyperplastic polyps and sessile serrated adenoma/polyps are also considered precancerous lesions [3,4], and guidelines of some Western countries recommend resecting these serrated polyps to prevent CRC [5]. Cold snare polypectomy (CSP) has been widely performed as a treatment for colorectal polyps < 10 mm in size owing to its safety and effectiveness [6,7]. One of the complications of CSP is bleeding that occurs immediately after resection (CSP-IB). CSP-IB interferes with endoscopic confirmation of the residual lesion, resulting in longer resection times. Several studies have reported on CSP-IB; however, its definition in different reports varies [8-13]. Moreover, to the best of our knowledge, only one prospective study focused on CSP-IB [9], and detailed data such as the frequency of CSP-IB occurrence and duration of bleeding have not yet been clarified. Submucosal injection with epinephrine-added solution is widely used to prevent bleeding and colonic perforation during conventional hot endoscopic resection. The mechanism of its hemostatic effect is thought to be due to the compression of blood vessels by the submucosal swelling caused by the injection and vasoconstriction effect of epinephrine. Submucosal injection is also administered during the CSP, and its advantages are as follows: it clearly delineates the margins of the lesion [14], the injection appears to make tissue transection easier, and bleeding is typically minimal [15]. Moreover, following a literature search, no randomized controlled trials have reported the CSP-IB suppression effect of submucosal injection with epinephrine-added saline in CSP (CEMR) in comparison with conventional CSP. In this study, we investigated whether CEMR saves the time required for the resection procedure and shortens the time to cessation of CSP-IB with CEMR compared to that with conventional CSP.

## Materials and methods

Study design

This study was a prospective, open-label, randomized controlled trial. It was conducted in accordance with the guidelines laid down in the Declaration of Helsinki and was approved by the Showa University Review Board (Registration No. 2089). The study was conducted at the Showa University Hospital from April 2019 to April 2021. Written informed consent for this study was obtained from all the participants before the procedure by one colonoscopy examiner (A.K.).

Study population

The inclusion criteria were patients aged 20-79 years undergoing colonoscopy. The exclusion criteria were 1) inflammatory bowel disease, 2) Lynch syndrome, 3) familial adenomatous polyposis, 4) active hematochezia, 5) colonic stenosis, 6) colonic diverticulitis, 7) mega-colon, 8) inadequate bowel preparation, 9) patients with incomplete total colonoscopy, 10) patients who had not discontinued antithrombotic drugs for an appropriate period [16], 11) those who refused to undergo polypectomy, and 12) patients with severe psychological and/or physical disease.

Each participant was randomized using an envelope method either to the CSP with submucosal injection (CEMR group) or conventional CSP (CSP group) when at least one polyp ≤ 10 mm in size was detected and diagnosed as a low-grade adenoma or a serrated polyp without dysplasia during colonoscopy. When an eligible lesion was detected, a nurse not involved in the study promptly removed one envelope from the box and opened it. The allocated group was written on the paper in the envelope, and the examiner was informed about the same. In cases with multiple polyps, all eligible polyps were resected using the assigned method.

Procedure

All participants underwent bowel preparation with 2 L of polyethylene glycol with ascorbic acid (Moviprep; Ajinomoto Pharmaceutical Co, Tokyo, Japan) before colonoscopy. One experienced colonoscopist performed all endoscopic procedures in this study. All colonoscopies were mainly performed with CF-HQ290Z or PCF-H290ZI (Olympus Medical Systems, Tokyo, Japan), and PCF-PQ260L was used instead of these scopes when cecal insertion was difficult. Black hood (MAJ-2187 or MAJ-2257; Olympus Medical Systems) was attached to the tip of the colonoscope during colonoscopic examination in all cases. Carbon dioxide insufflation was used throughout the colonoscopy procedure. The macroscopic features of the polyps were determined according to the Paris classification [17], and the size of the lesions was measured in comparison with a sheath of the snare or the tip of the injection needle. Endoscopic diagnosis of the detected lesions was initially determined using narrow-band imaging (NBI) according to the Japan NBI Expert Team (JNET) classification [18] with a magnifying colonoscope or NBI International Colorectal Endoscopic (NICE) classification [19] with a non-magnifying colonoscope (PCF-PQ260). If the lesion was suspected to be high-grade dysplasia or invasive cancer (JNET Type-2B, 3 or NICE Type 3), it was considered as an invalid lesion. The time to diagnosis and resection of the invalid lesion was excluded from the withdrawal time.

**Figure 1:**
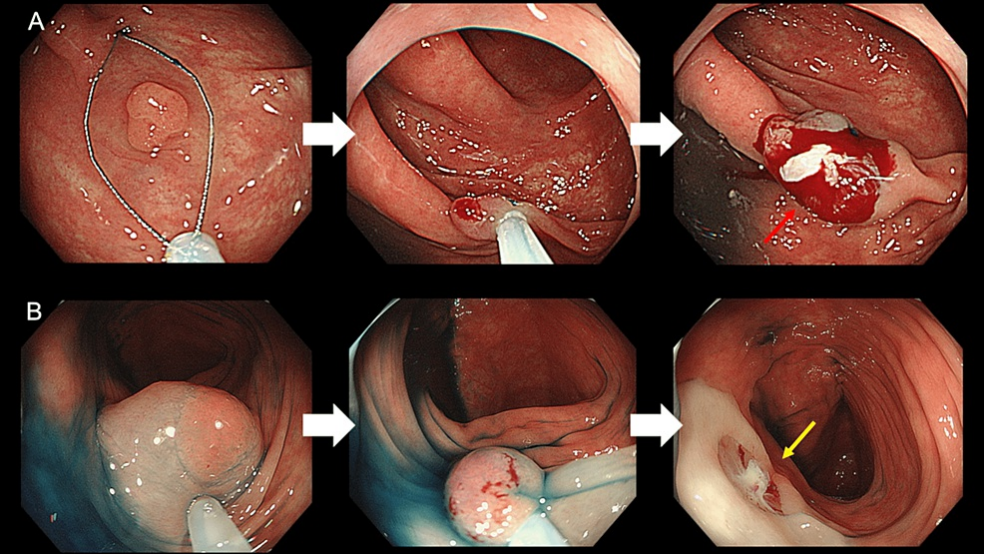
Endoscopic images of conventional CSP and CEMR A: A dedicated snare was fully opened to completely resect the lesion. CSP-IB occurred after CSP (red arrow). B: Cold endoscopic mucosal resection. Epinephrine (0.001%)-added saline was injected immediately before CSP. CSP-IB was suppressed after resection (yellow arrow). CEMR: Cold snare polypectomy with epinephrine-added submucosal injection CSP: Conventional cold snare polypectomy

All eligible lesions were resected with CSP using a dedicated snare with a diameter of 10 mm when opened (SD-400-U10, SnareMaster plus; Olympus Medical Systems). All lesions were ensnared with a fully opened snare, regardless of the polyp size, for complete excision. Epinephrine (0.001%)-added saline was injected immediately before CSP in the CEMR group (Figure 1).

The spontaneous cessation of CSP-IB, including oozing, was evaluated by an endoscopic nurse not involved in the procedure. After the cessation of CSP-IB, the CSP site was inspected endoscopically for residual lesions. If residual lesions were suspected, additional CSP was performed. Endoscopic hemostasis (e.g., clipping, thermal coagulation, and injection technique) was not allowed unless the examiner considered it absolutely necessary, and it was documented in the colonoscopic finding report if any hemostasis was performed. Resected specimens were retrieved through a scope channel into a polyp trap system. After each procedure, the obtained specimens were fixed with formalin and reviewed by experienced pathologists who were blinded to the patient information. Approximately two weeks after CSP, the patients visited our hospital and were enquired about any post-CSP complications (e.g., bloody stool, continuous abdominal pain, and fever).

Outcomes

The primary study outcome was the time required for resection (TR) (per lesion) between the two groups. TR was defined as the time from the initiation of resection (the first insertion of the snare in the CSP group or the injection needle in the CEMR group) to the end of resection (confirming complete resection endoscopically with NBI observation after recognizing the cessation of CSP-IB) in each lesion. The secondary outcome was the time required to CSP-IB (TIB), defined as the time from ensnaring the lesion to confirming the spontaneous cessation of CSP-IB. Still images were taken at the time of initiation of resection, ensnaring the lesion, confirmation of cessation of CSP-IB, and confirmation of complete resection. After each procedure, TR and TIB were calculated by referring to the time recorded in the still images. In the lesions where multiple attempts at ensnaring were required, all time durations, including those for multiple ensnaring, were summed while calculating the TR and TIB. A subgroup analysis was performed to identify the characteristics of the lesion wherein TR and TIB were reduced by the use of CEMR compared with conventional CSP. These included the size of lesion (< 5 mm vs. ≥ 5 mm), lesion morphology (protruded vs. flat), and lesion location (right colon vs. left colon).

Sample size calculation

The sample size was calculated based on the data from our previous retrospective study (Table 1).

**Table 1:** Results of our retropsective study (Supplement) SD: Standard deviation CEMR: Cold snare polypectomy with epinephrine-added submucosal injection CSP: Conventional cold snare polypectomy TR: Time requred for resection TIB: the time required to cessation of immediate bleeding

	Index	CEMR	CSP	Total
		n, (%)	n, (%)	n, (%)
No. of lesion		18	25	43
size	mm, median	5	4	4
Macroscopic type	Protruded	10 (55.5)	13 (52.0)	23 (53.5)
	Flat	8 (44.5)	12 (48.0)	20 (46.5)
Location	Right-side	14 (77.8)	12 (48.0)	26 (60.5)
	Left-side	4 (22.2)	13 (52.0)	17 (39.5)
Historogy	Low-grade adenoma	15 (83.3)	23 (92.0)	38 (88.4)
	Serrated lesion	3 (16.7)	1 (4.0)	4 (9.3)
	Non-neoplastic	0 (0)	1 (4.0)	1 (2.3)
TR	s, mean ± SD	104.1 ± 22.3	132.0 ± 60.2	120.3 ± 49.7
TIB	s , mean ± SD	34.3 ± 15.9	94.4 ± 52.0	69.3 ± 50.5

We hypothesized that the CEMR technique would reduce the TR by 40 s compared to the conventional CSP technique. We also assumed that one eligible lesion would be detected per patient. For detecting a difference at a two-sided alpha of 0.05, with a power of at least 90%, we estimated that at least 50 patients were required in each group. Considering a 20% deviation, we determined a sample size of 64 patients in each group, and a total of 128 patients were required.

Randomization

Participants were allocated to the CEMR group or the CSP group using the envelope method in a ratio of 1:1. This was not a double-blinded study because the examiner and procedural assistant could not perform CSP without knowing the allocation or looking at the endoscopic monitor, and all the participants were not barred from seeing the monitor.

Statistical analyses

Data are presented as the mean (standard deviation) or number (%). The groups were compared using repeated-measures analysis of variance to evaluate the primary and secondary outcomes. Post-hoc analysis was performed based on the size, morphology, and location of the lesion. The chi-squared test or Fisher’s exact test was performed on categorical data, and the Mann-Whitney U test was performed on continuous data for comparison between the two groups, when appropriate. All analyses were performed using JMP® 15 (SAS Institute Inc., Cary, NC, USA).

## Results

Participants

**Figure 2:**
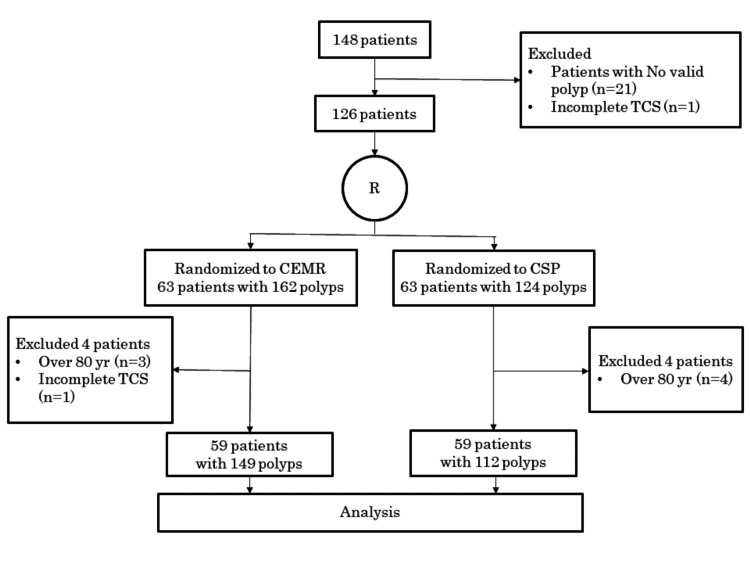
Flow diagram of the patient recruitment TCS: Total colonoscopy CEMR: Cold snare polypectomy with epinephrine-added submucosal injection CSP: Conventional cold snare polypectomy

The recruitment flowchart is shown in Figure 2.

Between June 2019 and April 2021, 151 patients were recruited, and 148 patients were enrolled in the study. Twenty-one patients without polyps or with lesions but no target lesions and one who did not undergo total colonoscopy were excluded from the analysis. Thus, a total of 126 patients were randomized; 63 patients were allocated to the CEMR group, and 63 patients to the CSP group. Four patients in the CEMR group and four in the CSP group were excluded because they were identified to be ineligible after treatment allocation. One patient in the CEMR group was randomized, even though insertion to the cecum was not performed. Three patients in the CEMR group and four patients in the CSP group were more than 80 years of age. In the remaining 118 patients, a total of 261 targeted lesions resected using CSP (149 lesions in 59 patients in the CEMR group and 112 lesions in 59 patients in the CSP group) were finally analyzed.

Baseline data of participants and polyps

The mean (± standard deviation [SD]) age of the patients in the study was 65.8 (± 12.0) years. Most colonoscopies were performed for surveillance post-polypectomy (54.2%) or positive fecal blood tests (20.3%). Moreover, 20 (16.9%) patients were on anticoagulant drugs (terminated for an appropriate period before the colonoscopy in compliance with guidelines for gastroenterological endoscopy in patients undergoing antithrombotic treatment: 2017 Appendix on Anticoagulants Including Direct Oral Anticoagulants [19]). The proportion of patients who underwent colonoscopy under the use of midazolam, pethidine hydrochloride, or both was 91.5% of the total cohort: 94.9% in the CEMR group, and 88.1% in the CSP group. The proportions of patients with excellent bowel cleansing were 76.3%, 71.2%, and 81.4% in the total cohort, CEMR group, and CSP group, respectively. The mean (± SD) cecal intubation time was 5.8 (± 3.1) min, 6.2 (± 3.5) min in the CEMR group, and 5.5 (± 2.6) min in the CSP group. The mean (± SD) withdrawal time was 11.9 (± 3.1) min in the total cohort, 12.6 (± 3.1) min in the CEMR group, and 11.2 (± 2.9) min in the CSP group. The mean (± SD) number of resected valid polyps per patient was 2.6 ± 1.9 in the CEMR group and 1.9 ± 1.3 in the CSP group. The baseline characteristics of the patients in both groups are shown in Table 2

**Table 2:** Patient characteristics SD: Standard deviation FOBT: Fecal occult blood test CEMR: Cold snare polypectomy with epinephrine-added submucosal injection CSP: Conventional cold snare polypectomy

	CEMR (n = 59)	CSP (n = 59)	Total (n = 118)
Age, mean (± SD), y	67.9 ± 10.2	63.7 ± 13.3	65.8 ± 12.0
Sex, No. (%)			
Male	33 (55.9)	36 (61.0)	69 (58.5)
Female	26 (44.1)	23 (39.0)	49 (41.5)
Antithrombotic drug, No. (%)			
No	48 (81.4)	50 (84.7)	98 (83.1)
Yes	11 (18.6)	9 (15.3)	20 (16.9)
Indication, No. (%)			
Surveillance	34 (57.6)	30 (50.9)	64 (54.2)
Positive FOBT	15 (25.4)	9 (15.2)	24 (20.3)
For resection	5 (8.5)	4 (6.8)	9 (7.6)
Screening	3 (5.1)	9 (15.2)	12 (10.2)
Symptomatic	2 (3.4)	7 (11.9)	9 (7.6)
Sedation, No. (%)			
Yes	56 (94.9)	52 (88.1)	108 (91.5)
No	3 (5.1)	7 (11.9)	10 (8.5)
Bowel preparation, No. (%)			
Excellent	42 (71.2)	48 (81.4)	90 (76.3)
Good	17 (28.8)	11 (18.6)	28 (23.7)
Time to insertion, (± SD), min.	6.2 ± 3.5	5.5 ± 2.6	5.8 ± 3.1
Time to withdrawal, (± SD), min.	12.6 ± 3.1	11.2 ± 2.9	11.9 ± 3.1
Number of resected valid polyp, No. (%)	2.6 ± 1.9	1.9 ± 1.3	2.3 ± 1.6

The mean (± SD) size of the lesions was 4.3 (± 1.8) mm. A total of 181 (69.3%) polyps were located in the right colon (cecum, ascending colon, and transverse colon), and 81 (30.7%) polyps were located in the left colon (descending colon, sigmoid colon, and rectum). The characteristics of the targeted lesions were similar in both groups (Table 3).

**Table 3:** Lesion characteristics SD: Standard deviation CEMR: Cold snare polypectomy with epinephrine-added submucosal injection CSP: Conventional cold snare polypectomy

	CEMR (n = 149)	CSP (n = 112)	Total (n = 261)	P value
Size,mean (± SD), mm	4.4 ± 1.8	4.2 ± 1.8	4.3 ± 1.8	0.186
Size, No. (%)				0.258
<5 mm	91 (61.1)	76 (67.9)	167 (64.0)	
≥5 mm	58 (38.9)	36 (32.1)	94 (36.0)	
Location, No. (%)				0.205
Right side	108 (72.5)	73 (65.2)	181 (69.3)	
Left side	41 (27.5)	39 (34.8)	80 (30.7)	
Morphology, No. (%)				0.198
Flat	98 (65.8)	82 (73.2)	180 (69.0)	
Protruded	51 (34.2)	30 (26.8)	81 (31.0)	
Pathology, No, (%)				0.188
Low-grade adenoma	110 (73.8)	86 (76.8)	196 (75.1)	
High-grade adenoma	1 (0.7)	0 (0)	1 (0.4)	
Hyperplastic polyp	29 (19.5)	13 (11.6)	42 (16.1)	
Sessile serrated adenoma/polyp	7 (4.7)	10 (8.9)	17 (6.5)	
Inflammatory polyp	1 (0.7)	3 (2.7)	4 (1.5)	
Loss of tissue	1 (0.7)	0 (0)	1 (0.4)	

Only one lesion in each group required two rounds of snaring.

Outcomes

The primary and secondary outcomes are shown in Table 4.

**Table 4:** Primary and secondary outcomes; Comparison between the two groups for the time required for resection and the time required to cessation of immediate bleeding The time required for resection calculated as the least square mean was significantly shorter in the CEMR group than in the CSP group, and the difference between the two groups was -24.6 s (95% CI -37.9 to -11.4 s). The time required to cessation of immediate bleeding was also significantly shorter in the CEMR group than in the CSP group, and the differences were -53.7 s (95% CI -62.7 to -44.8 s). CI: Confidence interval SD: Standard deviation CEMR: Cold snare polypectomy with epinephrine-added submucosal injection CSP: Conventional cold snare polypectomy

The time required for resection			
	Least square mean (s, 95% CI)	Difference (s, 95% CI)	P value
CEMR	106.3 (97.5, 115.4)	-24.6 (-37.9, -11.4)	<0.001
CSP	130.9 (121.2, 140.7)		
The time required to cessation of immediate bleeding			
	Least square mean (s, 95% CI)	Difference (95% CI)	P value
CEMR	20.4 (14.3, 26.5)	-53.7 (-62.7, -44.8)	<0.001
CSP	74.2 (67.6, 80.7)		

The TR calculated as the least square mean was significantly shorter in the CEMR group (106.3 s, 95% CI 97.5-115.4 s) than in the CSP group (130.9 s, 95% CI 121.2-140.7 s) (P < 0.001), and the difference between the two groups was -24.6 s (95% CI -37.9 to -11.4 s). The least-square mean TIB was also significantly shorter in the CEMR group than in the CSP group (20.4 s, 95% CI 14.3 to 26.5 s vs. 74.2 s 95% CI 67.6 to 80.7 s, P < 0.001), and the differences in TIB were -53.7 s (95% CI -62.7 to -44.8 s).

Post hoc analysis

**Figure 3:**
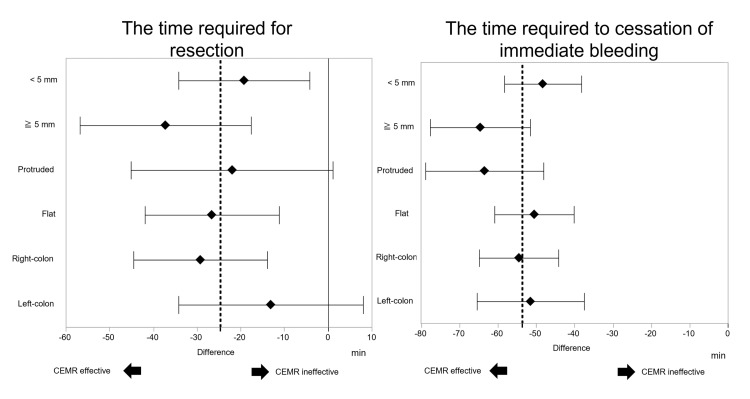
Forest plot of the difference in the time required for resection and the time required for spontaneous cessation of immediate bleeding between the two groups The dotted line shows the difference in the whole analysis. CEMR: Cold snare polypectomy with epinephrine-added submucosal injection

The subgroup analysis by characteristics of the lesion (size, morphology, and location) associated with TR and TIB is shown in Figure 3.

According to the size of the lesion, the difference in TR between the two groups was larger when the size of the lesion was ≥ 5 mm (-37.2 s, 95% CI -56.8 to -17.6 s) than when the size of the lesion was < 5 mm (-19.2 s, 95% CI -34.2 to -4.2 s) (p for interaction = 0.1346). On comparison by the location of the lesions, the difference in TR between the two groups was larger in the lesions located in the right colon (-29.2 s, 95% CI -44.5 to -13.9 s) than in these located in the left colon (-13.1 s, 95% CI -34.2 to 8.0 s) (p for interaction = 0.1941). A comparable result was noted in comparison with the morphology of the lesion (flat feature; -26.6 s, 95% CI -41.9 to -11.2 s and protruded feature; -21.9 s, 95% CI -45.1 to 1.1 s), and its interaction was smaller than that of the above conditions (p for interaction = 0.7312).

Complications

No lesions required hemostasis for CSP-IB. Nausea after colonoscopy occurred in two (3.4%) patients in the CEMR group. Submucosal hematoma occurred immediately after the procedure in one (0.9%) lesion in the CSP group, and preventive hemostasis with an endoscopic clip was performed. No cases of perforation or delayed bleeding occurred in either group.

## Discussion

This prospective randomized trial demonstrated that CEMR is effective for reducing the TR. Moreover, CEMR significantly reduced the TIB. Compared with the conventional CSP method, the CEMR method requires insertion of the injection needle and submucosal injection, which would be expected to require more time. However, the CEMR group had a shorter mean TR of -24.6 s (95% CI -37.9 to -11.4 s) than the CSP group. The mean TIB was shorter by -53.7 s (95% CI -62.7 to -44.8 s) in the CEMR group than in the CSP group, and the effect of the decrease in the TIB by the submucosal injection might be the reason for the shorter TR. Several studies have described CSP-IB [8-13]. To the best of our knowledge, only one prospective study by Repici et al. investigated CSP-IB as the main outcome of the study and concluded that the rate of CSP-IB was 1.8% [9]. In their study, CSP-IB was defined as any immediate episode requiring any form of endoscopic hemostasis. Other reports investigated the rate of CSP-IB as a secondary outcome. Horiuchi et al. and Aoki et al. defined CSP-IB as spurting or oozing of blood that continued for more than 30 s after resection and required hemostatic clipping [8,10]. In their study, the CSP-IB rates were 8.0% and 5.7%, respectively. Aizawa et al. reported that the rate of CSP-IB was 54.0%; they defined CSP-IB a spurting or oozing of blood that continued for more than 1 min [12]. In this study, the least-square mean TIB, which is considered almost equivalent to CSP-IB, was 95.8 s (95% CI 87.5 to 104.1 s) in the CSP group; 109 of 112 lesions (97.3%) and 88 of 112 (78.6%) lesions in this group required more than 30 s and 60 s, respectively, for the spontaneous cessation of CSP-IB. The CSP-IB rate in this study appears to be higher than that in other reports. We defined TIB as the time from ensnaring the lesion to the spontaneous cessation of immediate bleeding, and a very small amount of oozing was treated as continuous bleeding. Furthermore, the objective confirmation of spontaneous cessation of CSP-IB was performed by an endoscopic nurse not involved in the procedure. Aoki et al. reported the resection at a distance of 1 mm to a few mm from the polyp to maintain a non-neoplastic mucosal margin [8]. In this study, all lesions were ensnared with a fully opened snare, regardless of polyp size, for complete excision using a dedicated snare.

The difference in the proportion of lesions that developed CSP-IB between our study and previous reports might have been due to a difference in the definition of CSP-IB and a wider range of mucosal resection in this study.

In this study, no hemostatic clips were used, and hemostasis for CSP-IB was not performed in any cases. This result suggests that aggressive hemostasis might not be necessary even if CSP-IB occurs when antithrombotic drugs are discontinued before the procedure. Therefore, we believe that our study provides detailed insights into the management of CSP-IB.

In this study, the mean number of polyps per patient was higher in the CEMR group than in the CSP group (2.6 ± 1.9 vs. 1.9 ± 1.3). CSP-IB, which continued for a longer time in the CSP group than in the CEMR group, might have affected the visualization of the large intestine and made it difficult to detect other small polyps due to oozing blood. These findings suggest that CEMR might be particularly effective in patients with multiple polyps.

Post-hoc analysis showed that the reduction in TR/TIB was higher in lesions ≥ 5 mm than in lesions < 5 mm in size. This result suggests that CEMR might be highly effective for large lesions. Although residual confirmation may not be necessary in CSP with small colonic polyps, CSP-IB suppression by CEMR for residual confirmation may be useful in large lesions with high residual risk. A piecemeal CSP for colorectal lesions of ≥ 10 mm has also been attempted [20-22]; in such cases, the bleeding needs to be stopped to determine whether complete resection has been performed. The hemostatic effect of suppressing CSP-IB using the CEMR method might be useful for piecemeal CSP for large lesions as well.

The effect of CEMR in shortening the TR might be higher in lesions in the right colon than those in the left colon. The exact reason for the difference based on the location of the lesion is unknown. Serrated polyps are distributed predominantly in the right colon and have a low risk of advanced dysplasia, even in large lesions [23,24]. Serrated polyposis syndrome, which is characterized by the presence of multiple SPs, is associated with increasing CRC incidence risk [25,26]. For patients with serrated polyposis syndrome, complete removal of all polyps ≥ 3 mm can prevent CRC [27]. A study also reported that the therapeutic effect of piecemeal CSP for large SPs is equivalent to that of conventional EMR [22]. Thus, CEMR might also be particularly effective in patients with serrated polyposis syndrome.

A limitation of CEMR is the cost of the injection needle and epinephrine-added saline; these items are not required for performing conventional CSP. Performing CEMR may not be as necessary for patients with a small number of colorectal polyps as for those with multiple polyps. However, in patients with multiple polyps, shortening the TR allows for more polyps to be resected, which may shorten the examination time and reduce the number of colonoscopies. Therefore, this method is considered to be highly useful, especially for patients with multiple polyps despite these additional costs.

This study has some limitations. First, this was a single-center, open-label trial. A double-blind design was not suitable for this study because the allocated treatment method could not be blinded from the endoscopist, and the participants were not barred from looking at the endoscopic monitor. Second, although all colonoscopic procedures were performed by an experienced endoscopist, the duration of the resection procedure might vary depending on the endoscopist’s experience. Third, the treatment efficacy of CEMR compared with CSP was not evaluated. Shimodate et al. demonstrated that CSP with submucosal injection resulted in a lower rate of negative lateral and vertical margins than conventional CSP [13]. However, it is unclear whether a positive or unknown pathological margin in CSP specimens is associated with residual CSP. Pathological estimation of the margin for specimens obtained by CSP is difficult due to fragmentation caused during polyp retrieval through the scope channel [28]. In their systematic review and pooled analysis, Chandrasekar et al. reported residual polyp rates of 4.1% (95% CI, 0.2 to 8.4%) in conventional CSP and 4.7% (95% CI, 0.7 to 10.1%) in CEMR for polyps ≧ 10 mm [29]. Thus, we believe that the CEMR method does not reduce the therapeutic effect compared with the conventional CSP method.

## Conclusions

In conclusion, CSP with epinephrine-added saline submucosal injection decreased the time for resection compared with conventional CSP in colorectal polyps < 10 mm. The CEMR method was also effective in reducing the time required for the spontaneous cessation of CSP-IB. The safety of this method was high, and no delayed bleeding or colonic perforation was observed. Its usefulness might be even higher in patients with multiple polyps.
